# Modeling and analysis of psychological mechanism for preventive behaviors against the COVID-19

**DOI:** 10.1093/eurpub/ckac131.070

**Published:** 2022-10-25

**Authors:** Y Fukukawa, S Kurita

**Affiliations:** 1 Psychology, Waseda University, Tokyo, Japan; 2 Graduate School of Letters, Arts and Sciences, Waseda University, Tokyo, Japan

## Abstract

**Background:**

The COVID-19 pandemic has dramatically changed human life style. People all over the world are still on the way to establish “New Normal”. The purpose of this study was to construct a cognitive model that predicts preventive behaviors against the infectious disease.

**Methods:**

A total of 3,000 Japanese respondents aged from 18 to 86 years participated in a web survey in January 2021. The data of 2,913 respondents (1,633 males and 1,280 females) were eligible for analysis. The following information regarding cognitive characteristics was assessed: 1) Cognitive reflection ability (Frederick, 2005), 2) Experiential thinking style (Pacini & Epstein, 1999), 3) Germ aversion (Duncan et al., 2009). In addition to those characteristics, the participants were asked to answer 6 items to rate their preventive behaviors against the COVID-19, such as keeping social distance and wearing a mask. A structural equation modeling technique was used to test the causal relationships among the measures. We hypothesized that experiential thinking style and germ aversion would mediate the causal link between cognitive reflection ability and preventive behaviors.

**Results:**

Correlation analyses indicated that cognitive reflection ability was negatively associated with experiential thinking style, whereas experiential thinking style was negatively associated with germ aversion. Furthermore, the higher germ aversion was, the more the participants enforced the covid-19 preventive behaviors. Parameter estimation of the causal model using the measures by the bootstrap method indicated that the model acceptably fit the data with CFI of 0.997, TLI of 0.996, and RMSEA of 0.008 (95% CI = 0.004:0.011).

**Conclusions:**

The findings of the study suggest that there is a cognitive psychological process that induces preventive behaviors against the COVID-19. The results should be useful to improve public health interventions for future pandemics.

**Key messages:**

• A web survey was conducted for 3,000 Japanese adults to construct a cognitive model that predicts preventive behaviors against the COVID-19.

• The structural equation modeling indicated that the participants’ experiential thinking style and germ aversion mediated the causal link between cognitive reflection ability and preventive behaviors.

